# Body Composition and Kidney Outcomes: A Cohort Study of Rapid Kidney Function Decline and a Mendelian Randomization Analysis of CKD Incidence

**DOI:** 10.1016/j.xkme.2025.101087

**Published:** 2025-08-14

**Authors:** Kaixin Li, Yao Liu, Jiaxi Zhao, Zhibin Ye

**Affiliations:** 1 Department of Nephrology, Huadong Hospital, Fudan University, Shanghai, China; 2 Shanghai Key Laboratory of Clinical Geriatric Medicine, Huadong Hospital, Fudan University, Shanghai, China; 3 Department of General Practice, General Practice Medical Center, West China Hospital, West China School of Medicine, Sichuan University, Chengdu, Sichuan, China

**Keywords:** Appendicular lean mass index, body composition, body fat percentage, chronic kidney disease, muscle mass, rapid kidney function decline, sarcopenic obesity

## Abstract

**Rationale & Objective:**

Chronic kidney disease (CKD) is a global health issue, potentially arising from rapid kidney function decline (RKFD). Although body composition influences various metabolic disorders, its relationship with kidney outcomes remains unclear. This study aimed to investigate the impact of body composition on RKFD and CKD risk.

**Study Design:**

A cohort study assessed the relationship between body composition and RKFD. A 2-sample Mendelian randomization approach investigated genetic evidence linking body composition to CKD risk.

**Setting & Participants:**

In total, 229 adults aged 50-70 years with normal kidney function were recruited from Huadong Hospital in Shanghai, China.

**Predictors:**

Body composition indicators include fat mass, lean mass, appendicular lean mass index, fat mass index, total and regional body fat percentages, and sarcopenic obesity, defined by high body fat percentages (>27% in men, >38% in women) and low lean mass (appendicular lean mass index <7.26 kg/m^2^ in men, <5.45 kg/m^2^ in women).

**Outcomes:**

The primary outcome was RKFD. The second was genetically predicted CKD risk.

**Analytical Approach:**

Cox regression and subgroup analyses assessed observational associations. The Mendelian randomization study used two-sample Mendelian randomization, multivariable, and bidirectional Mendelian randomization.

**Results:**

RKFD occurred in 9.9% of participants. Lower appendicular lean mass index and the presence of sarcopenic obesity were associated with higher RKFD risk. In overweight participants and those with baseline estimated glomerular filtration rate >90 mL/min/1.73 m^2^, the negative association between appendicular lean mass index and RKFD remained significant. Mendelian randomization analysis revealed that genetically predicted legs and whole-body fat percentages increased CKD risk, whereas appendicular muscle mass was negatively associated with CKD.

**Limitations:**

Differences between the outcomes require further validation. Some sample overlap in the Mendelian randomization analysis may introduce bias.

**Conclusions:**

Lower appendicular lean mass index and sarcopenic obesity were associated with RKFD. Higher leg and whole-body fat percentages and lower appendicular muscle mass significantly contribute to CKD risk, highlighting the importance of body composition in kidney health.

## Plain Language Summary

Body composition, such as fat and muscle mass and percentage, may play a role in kidney outcomes. In this study, we followed a group of middle-aged to old adults with normal kidney function to see how body composition affects how quickly kidney function declines over time. We further explore whether genetically predicted fat percentages and lean mass in certain body parts can increase the risk of developing chronic kidney disease. We found that people with less muscle and with higher body fat percentage were more likely to experience rapid kidney function decline or develop kidney disease, suggesting body composition management may prevent kidney problems.

A major worldwide health problem, the prevalence of chronic kidney disease (CKD) steadily rising alongside the aging population.^1^ It has been established that CKD is associated with a number of risk factors, such as hypertension, diabetes mellitus, hyperlipidemia, smoking, and obesity.^2^ Notably, rapid kidney function decline (RKFD) can precipitate the onset and exacerbation of CKD. Individuals with type 2 diabetes who have risk factors, such as dyslipidemia, macroalbuminuria, and poor lipid and blood sugar management, may get severe diabetic kidney disease in a matter of months.^3^ RKFD has also been observed in individuals with normal kidney function, with reported incidence rates ranging from 2% to 17%.^4^^,^^5^ Although hypertension, metabolic syndrome, and obesity have emerged as noteworthy predictors of RKFD in the elderly population^5^, ^6^, ^7^, the risk factors for RKFD in the middle-aged to elderly population remain obscure.

**Figure 1:**
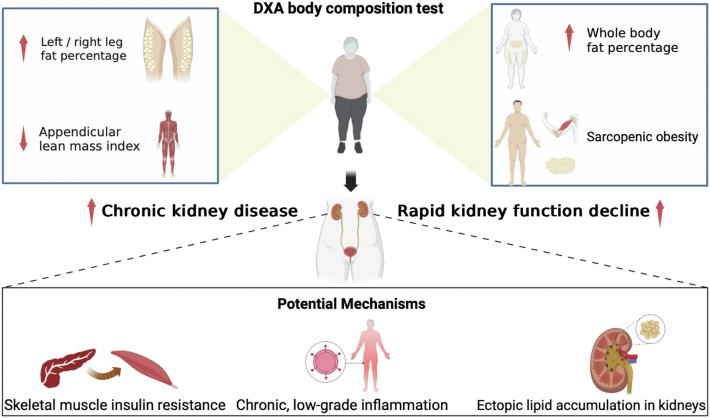
Body composition and incident chronic kidney disease and rapid kidney function decline.

Several epidemiological studies have shown a connection between higher body mass index (BMI) and increased risks of kidney disease.^8^ A Mendelian randomization (MR) study revealed a causal relationship between genetically higher BMI and CKD and key markers of kidney health such as estimated glomerular filtration rate (eGFR) and serum urea nitrogen.^9^ However, BMI, as a measurement of obesity, is flawed, because it does not take body composition or fat distribution into consideration. Although the distribution of body fat has been recognized as an essential factor in patients with metabolic dysfunction^10^, cardiovascular diseases^11^, and diabetic kidney disease^12^, limited evidence exists regarding the impact of fat distribution in individuals with normal kidney function. As our understanding of obesity continuously evolves, it has been shown that lean mass and fat percentage are closely associated with various metabolic diseases, notably cardiovascular disease mortality and the onset of diabetes.^13^ Furthermore, other research has proposed that body composition status should be considered a significant risk factor for RKFD.^5^ Therefore, the purpose of this study was to explore the relationship between body composition and CKD and RKFD (Fig 1). We conducted a cohort study to observe the Chinese middle-aged to elderly population without kidney disease, and we further carried out MR analyses investigating the causal effect of fat percentage and appendicular muscle mass on CKD using summary data from large-scale genome-wide association studies (GWAS) in the European population.

## Methods

### Cohort Study

#### Participants

**Figure 2:**
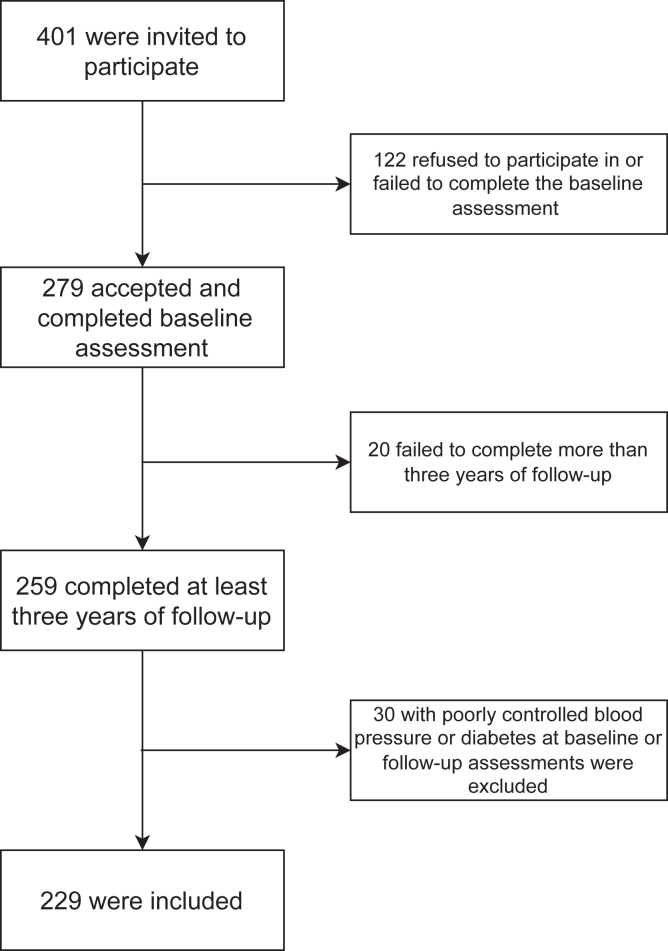
The flowchart for recruitment of cohort study.

This cohort study included 229 adults aged 50-70 years with normal kidney function, recruited from Huadong Hospital in Shanghai, China. In total, 401 individuals were invited to participate, and 279 completed baseline assessments. In total, 259 participants had completed at least 3 years of follow-up. Of these, 229 met the eligibility criteria and were included in the final analysis (Fig 2). All participants had an eGFR ≥60 mL/min/1.73 m^2^ and had no proteinuria or hematuria at recruitment. Annual health assessments (2010-2022) included body composition measurements and blood and urine tests. Exclusion criteria included conditions that could significantly affect kidney function, such as glomerular or tubular diseases, severe infections with sepsis, and nephrotoxic medications. Individuals with prior kidney transplants or kidney replacement therapy were excluded, as were those with poorly controlled blood pressure (≥130 mm Hg or diastolic blood pressure ≥80 mm Hg) or diabetes (HbA1c ≥7%) at baseline or follow-up. Participants with conditions affecting body composition (eg, long-term immobility or malnutrition, systemic corticosteroids) were also excluded. The study was approved by the Ethics Committee of Huadong Hospital (20240094).

#### Body Composition Measurement

Body composition was assessed using dual-energy X-ray absorptiometry.^14^ We measured parameters such as fat mass (FM), lean mass (LM), appendicular LM index (ALMI), and fat mass index. Measurements included segmental assessments of both arms, both legs, and trunk, as well as total masses derived from segmental addition. Fat percentages were computed as the ratio of fat masses to the total mass of either the entire body or specific body regions. ALMI was calculated by adding the LM of both arms and legs normalized for squared height and fat mass index was calculated by total fat mass normalized for squared height.

#### Assessment of Kidney Function

Serum creatinine levels were measured at baseline and during annual follow-up assessments. The eGFR was calculated using the Chronic Kidney Disease Collaboration (CKD-EPI) 2021 equation.^15^ RKFD was defined as a decline in eGFR > 3 mL/min/1.73 m^2^ per year during follow-up.^16^ The annual eGFR decline rate was calculated as (baseline eGFR – latest eGFR)/follow-up time.

#### Other Measurements

BMI was weight (kg)/height (m)^2^. Diabetes was defined as undergoing glucose-lowering therapy. Hypertension was defined as having hypertensive treatment or systolic blood pressure ≥ 140 mm Hg or diastolic blood pressure ≥ 90 mm Hg.^17^ Insulin resistance was quantified using the Homeostasis Model Assessment-Insulin Resistance and was calculated as fasting insulin (μIU/mL) × fasting glucose (mg/dL)/405.^18^ High body fat percentage (BF%) was determined as BF% > 27% in men and 38% in women. Low LM was defined as ALMI < 7.26 kg/m^2^ in men and < 5.45 kg/m^2^ in women. Sarcopenic obesity (SO) was defined as the coexistence of obesity and low LM.^19^^,^^20^

### MR Analysis

#### Genetic Instruments for Body Composition

We extracted GWAS summary data of fat percentages (n = 331,296) in a UK Biobank study^21^ from the IEU openGWAS database^22^, which estimated body composition using impedance measurement, and units of measurement are percent. The appendicular muscle mass data were obtained from the National Human Genome Research Institute–European Bioinformatics Institute Genome-Wide Association Studies (NHGRI-EBI GWAS) Catalog in European Populations (Study No. GCST90000025) with a sample size of 450,243.^23^ Single-nucleotide polymorphisms (SNPs) strongly associated with exposures (*P* < 5 × 10^-8^) were used as instrumental variables (IVs). For reverse MR analysis, CKD-associated SNPs were selected using a more relaxed threshold (*P* < 5 × 10^-6^), which had been used in previous MR research. To ensure exposure instrument independence, linkage disequilibrium SNPs were estimated (r2 0.001, clumping window = 10,000 kb). *F* statistics were used to assess IV strength.^24^

#### GWAS Summary Data of CKD

The outcome summary statistics for CKD, defined as impaired kidney function resulting from chronic kidney damage persisting for 3 or more months, were obtained from the fifth release of the FinnGen.^25^

#### MR Analysis

Two-sample Mendelian Randomization analysis^26^ were conducted using inverse-variance weighted (IVW), MR-Egger, and weighted median. The IVW method was selected as the primary technique. Multiple correction testing was addressed using the false discovery rate (FDR) adjusted *P* values proposed by Benjamini and Hochberg.^27^ If FDR is lower than 0.05, the result indicates statistical significance and suggests evidence of a causal relationship.

#### Sensitivity Analysis

Cochran's Q test, the MR-Egger regression test, and the Mendelian Randomization Pleiotropy Residual Sum and Outlier (MR-PRESSO) test identified heterogeneity or pleiotropy.^28^, ^29^, ^30^ A “leave-one-out” strategy was used to check reproducibility.

#### Multivariable MR

Multivariable MR (MVMR) was applied to estimate the effect of multiple exposures on a single outcome using genetic variants associated with potentially connected exposures.^31^

#### Statistical Analysis

In the cohort study, participants were grouped by RKFD status. Continuous variables were summarized using means with standard deviations or median with interquartile range. The categorical variables were expressed as percentages. Baseline differences between the RKFD and non-RKFD groups were calculated by *t* test for continuous variables and normally distributed data, Mann–Whitney U test for nonnormally distributed data, and χ^2^ test for categorical data. To analyze the association between body composition and RKFD, we employed a staged Cox regression analysis. Initially, the one-factor Cox regression was performed with each indicator. Subsequently, a comprehensive multivariable Cox regression model was constructed, including the indicators with *P* < 0.1 in the one-factor Cox regression. To further ascertain the independent impacts of indicators, separate multivariable Cox regression models were employed, each including the body composition indicator alongside the same set of well-established risk factors for RKFD, including age, sex, BMI, baseline eGFR, hypertension, diabetes, and hyperuricemia. In this assessment, fat mass index, ALMI, whole-body LMI, and BF% were considered continuous variables, while low muscle mass, high body fat percentage, and SO were considered categorical variables. Results were reported as hazard ratio with 95% confidence interval (CI). Subgroup analyses were performed based on sex, baseline kidney function, and BMI. A *P* value < 0.05 was considered statistically significant.

Cohort analysis was carried out using SPSS (version 26). All statistical analyses of the MR study were performed by the tow-sample MR package (version 0.5.6^32^) and MR-PRESSO package (version 1.0) in R (version 4.2.1). The STROBE-MR checklist for reporting MR studies was used in this study.^33^

## Results

### Baseline Characteristics and Incident RKFD

The participants in this study had an average age of 58.78 years, a mean baseline eGFR of 95.65 mL/min/1.73 m^2^, and a mean annual decline rate of 0.68 mL/min/1.73 m^2^. The incidence of RKFD was observed in 9.9% of the cohort. A detailed presentation of the baseline characteristics for participants with and without RKFD is provided in Table 1.

**Table 1:** Baseline Characteristics of the Study Population

Variables	Without RKFD	With RKFD	T/F	*P* Value
Number of participants, n	206	23		
Male, % (n)	154 (74.8%)	10 (43.5%)	10.07	0.002^a^
Age, y	58.5 ± 5.14	60.22 ± 7.32	–1.09	0.74
Beseline eGFR (mL/min/1.73 m^2^)	95.5 ± 10.67	97.20 ± 6.73	–0.75	0.46
BMI, kg/m^2^	24.98 ± 2.62	24.19 ± 3.20	1.34	0.18
Body composition				
FMI (kg/m^2^)	7.84 ± 1.36	8.42 ± 1.75	–1.88	0.14
ALMI (kg/m^2^)	6.88 ± 1.00	6.06 ± 1.12	3.37	<0.001^a^
LMI (kg/m^2^)	16.60 ± 2.13	15.58 ± 2.53	2.12	0.04^a^
Total body fat mass (g)	22,945.94 ± 4,005.93	21,949.65 ± 4,095.71	1.13	0.26
Total body fat percentage (%)	31.27 ± 4.55	33.22 ± 5.49	–1.92	0.06
Trunk fat percentage (%)	32.65 ± 4.38	33.68 ± 5.24	–1.05	0.30
Hip fat percentage (%)	31.16 ± 5.55	34.13 ± 5.98	–2.37	0.02^a^
Left arm fat mass (g)	1,290.82 ± 279.66	1,252.96 ± 300.19	0.59	0.55
Left arm fat percentage (%)	31.80 (28.20, 38.85)	34.3 (29.35, 43.21)	–1.81	0.07
Right arm fat mass (g)	1,319.4 ± 286.31	1,311.91 ± 338.43	0.10	0.92
Right arm fat percentage (%)	29.70 (26.65-36.15)	31.15 (26.95-41.80)	–1.65	0.10
Left leg fat mass (g)	3,314.37 ± 712.63	3,394.00 ± 720.73	–0.51	0.61
Left leg fat percentage (%)	29.90 ± 6.16	33.71 ± 6.90	–2.78	0.006^a^
Right leg fat mass (g)	3,396.50 ± 739.36	3,493.87 ± 709.41	–0.60	0.55
Right leg fat percentage (%)	30.05 ± 6.25	33.66 ± 7.22	–2.59	0.01^a^
Total body fat mass excluding head (g)	21,651.78 ± 3,945.24	20,801.19 ± 3,956.75	0.999	0.32
Total body fat percentage excluding head (%)	31.83 ± 4.88	34.16 ± 5.87	–2.165	0.03^a^
High BF, n (%)	134 (65.0%)	17 (73.9%)	0.72	0.49
Low LM, n (%)	98 (47.6)	18 (78.3)	7.80	0.005^a^
SO, n (%)	72 (35.0%)	14 (60.9%)	5.93	0.02^a^
SUN (mmol/L)	5.73 ± 2.89	5.77 ± 1.32	–0.07	0.94
Blood creatinine (umol/L)	74.45 ± 13.85	68.85 ± 11.05	1.87	0.06
CRP (mg/L)	0.64 (0.39-1.36)	1.11 (0.49-1.51)	–1.43	0.15
ESR (mm/h)	10.23 ± 10.60	14.04 ± 9.54	–1.65	0.10
Total cholesterol (mmol/L)	4.61 ± 0.91	4.94 ± 1.09	–1.60	0.11
Triacylglycerol (mmol/L)	1.40 (1.00-2.00)	1.25 (1.10-1.87)	–0.16	0.88
HDL (mmol/L)	2.08 ± 10.78	1.27 ± 0.31	0.36	0.72
LDL (mmol/L)	2.66 ± 0.77	2.85 ± 0.88	–1.09	0.28
HbA1c (%)	5.69 ± 0.38	5.58 ± 0.38	1.21	0.23
Fasting blood glucose (mmol/L)	5.55 ± 0.63	5.33 ± 0.49	1.58	0.12
Blood glucose 2 hours after meal (mmol/L)	8.76 ± 5.29	7.44 ± 1.77	1.16	0.25
Fasting C-peptide (ng/mL)	2.48 ± 1.03	2.13 ± 0.84	1.55	0.12
C-peptide 2 hours after meal (ng/mL)	9.51 ± 8.74	8.60 ± 5.03	0.48	0.63
Fasting insulin (mIU/L)	12.82 ± 12.28	11.59 ± 5.13	0.47	0.64
Insulin 2 hours after meal (mIU/L)	51.60 (31.00-87.35)	50.15 (26.85-98.05)	–0.64	0.52
HOMA-IR	3.22 ± 3.33	2.67 ± 1.46	0.78	0.44
Mean heart rate per min	68.65 ± 7.36	67.05 ± 6.99	0.91	0.37
Mean systolic blood pressure (mm Hg)	110.92 ± 12.62	113.95 ± 9.73	–1.01	0.31
Mean diastolic blood pressure (mm Hg)	68.21 ± 9.42	69.21 ± 7.84	–0.44	0.66
Hypertension, n (%)	77 (37.4%)	11 (47.8%)	0.95	0.37
Glucose metabolism				
Insulin resistance, n (%)	103 (50.7%)	13 (56.5%)	0.28	0.66
Elevated fasting glucose, n(%)	90 (43.7%)	7 (30.4%)	1.49	0.27
DM, n (%)	33 (16.0%)	2 (8.7%)	0.86	0.54
Hyperuricemia, n (%)	57 (27.7%)	6 (26.1%)	0.03	1.00

Abbreviations: BMI, body mass index; ALMI, appendicular lean mass index; LMI, lean mass index; SO, sarcopenic obesity; LM, lean mass; BF, body fat; SUN, serum urea nitrogen; DM, diabetes mellitus; eGFR, estimated glomerular filtration rate; HbA1C, glycated hemoglobin; HOMA-IR, homeostatic model assessment-insulin resistance; HDL, high-density lipoprotein; CRP, C-reactive protein; LDL, low-density lipoprotein; ESR, erythrocyte sedimentation rate; RKFD, rapid kidney function decline; DM, diabetes mellitus.

a Indicates statistical significance at *P* < 0.05.

The RKFD group has a lower percentage of males, lower ALMI, lower LMI, higher whole-body fat percentage (except the head), higher hip fat percentage, higher left leg fat percentage, higher right leg fat percentage, a higher prevalence of low LM, and a higher prevalence of SO.

### Association Between Body Composition and RKFD

The association between body composition elements and the risk of incident RKFD is displayed in (Table S1). Univariate Cox regression analysis revealed that an increased percentage of whole-body fat, a decreased ALMI, elevated hip and leg fat percentages, and the presence of SO were associated with an elevated risk of RKFD.

To ensure comprehensive factor inclusion, variables with *P* values below 0.1 in the univariate analysis were incorporated into subsequent multivariable regression models (Table 2). According to the multivariable Cox regression analysis, ALMI and the prevalence of SO along with whole-body fat percentage were statistically significantly associated with RKFD. Notably, the direction of the hazard ratio for whole-body fat percentage and RKFD reversed in this analysis.

**Table 2:** Association Between Body Composition and RKFD in Multivariable Cox Regression

Variables	HR	CI	*P* Value
Hip fat percentage (%)	1.043	0.733-1.486	0.81
FMI (kg/m^2^)	1.662	0.952-2.900	0.07
ALMI (kg/m^2^)	0.311	0.155-0.625	0.001
Left arm fat percentage (%)	0.990	0.796-1.232	0.93
Right arm fat percentage (%)	1.108	0.901-1.363	0.33
Left leg fat percentage (%)	1.008	0.716-1.418	0.10
Right leg fat percentage (%)	1.139	0.791-1.639	0.49
SO	2.890	1.003-8.326	0.05
Whole-body fat percentage excluding head (%)	0.610	0.385-0.966	0.04

Abbreviation: FMI, fat mass index; HR, hazard ratio; ALMI, appendicular lean mass index; SO, sarcopenic obesity.

**Figure 3:**
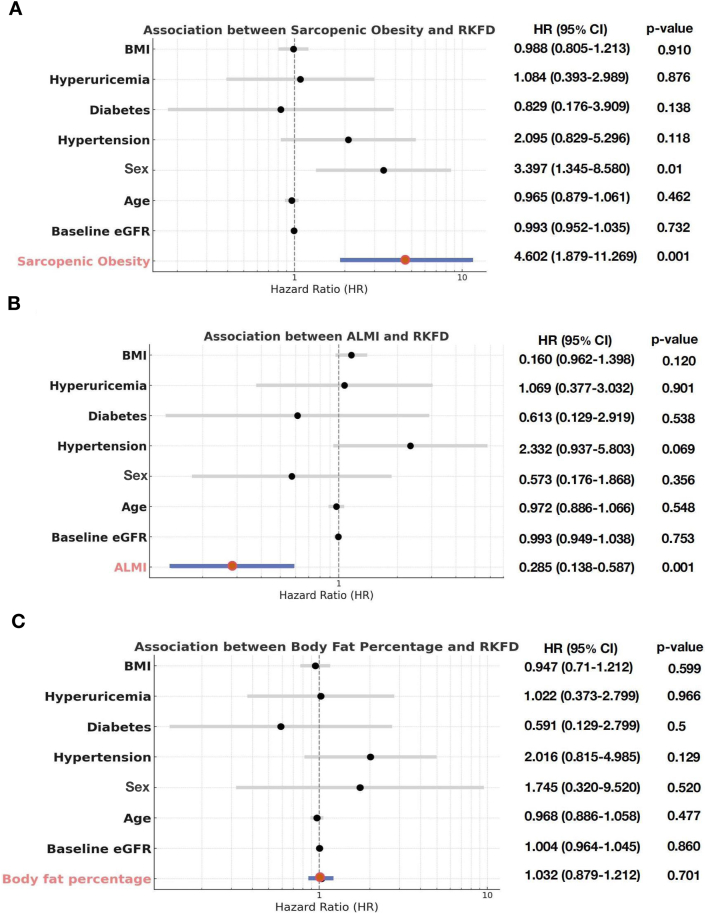
The independent effects of ALMI, whole-body fat percentage, and SO on RKFD. (A) The association between SO and RKFD. (B) The association between ALMI and RKFD. (C) The association between whole-body fat percentage and RKFD. The Cox regression models adjusted for BMI, hyperuricemia, diabetes, hypertension, sex, age, and baseline eGFR. ALMI, appendicular lean mass index; BMI, body mass index; eGFR, estimated glomerular filtration rate; SO, sarcopenic obesity; RKFD, rapid kidney function decline.

We then conducted 3 separate multivariable Cox regression models to delineate the independent effects of ALMI, whole-body fat percentage, and SO on RKFD, controlling for other established risk factors for kidney disease (Fig 3). After adjusting for confounding factors, each unit increase in ALMI was associated with a significantly lower risk of RKFD (hazards ratio, 0.285; 95% CI, 0.138–0.587; *P* = 0.001), and SO (hazards ratio, 4.602; 95% CI, 1.879–11.269, *P* = 0.001) continued to be associated with a higher risk of incident RKFD. However, the relationship between whole-body fat percentage and RKFD was no longer observed (hazards ratio, 1.032; 95% CI, 0.879–1.212; *P* = 0.70).

### Subgroup Analysis of ALMI and RKFD

**Figure 4:**
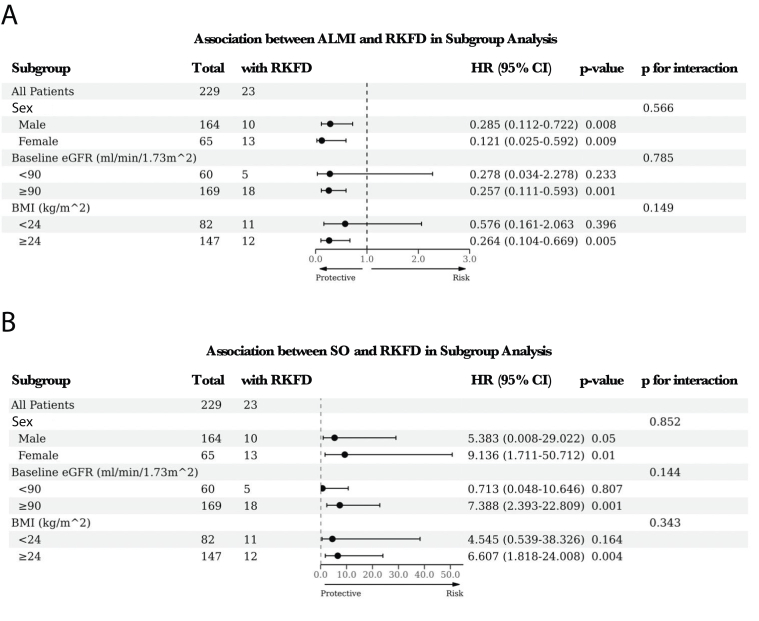
Subgroup analysis of the association between ALMI, RKFD, and RKFD. (A) The association between ALMI and RKFD in subgroup analysis. (B) The association between SO and RKFD in subgroup analysis. Subgroup analyses were performed based on sex, baseline kidney function, and BMI. ALMI, appendicular lean mass index; BMI, body mass index; SO, sarcopenic obesity; RKFD, rapid kidney function decline.

To further explore the relationship of ALMI and SO with RKFD in different kinds of people, a subgroup analysis was conducted, and the results are shown in Figure 4. After adjusting for confounding factors, it was observed that sex did not modify the association between ALMI and RKFD risk. However, the relationship between SO and RKFD was found to be significant exclusively in female participants. In the context of baseline kidney function, significant associations between ALMI and RKFD, as well as SO and RKFD, were observed in individuals with a baseline eGFR ≥ 90 mL/min/1.73 m^2^, but not in those with a baseline eGFR ≤ 90 mL/min/1.73 m^2^. Additionally, ALMI and SO showed significant links with RKFD in participants classified as overweight (BMI ≥ 24 kg/m^2^). Conversely, these associations were not evident in those with a BMI less than 24 kg/m^2^.

### Primary MR for CKD Risk Factors

To fulfill an essential assumption of MR analysis, which requires genetic instruments to be associated with the risk factor of interest, we removed the SNPs with F < 10 of each exposure. Finally, we obtained 202 SNPs for right leg fat percentage, 206 for left leg fat percentage, 201 for right arm fat percentage, 213 for left arm fat percentage, 195 for trunk fat percentage, and 190 for whole-body fat percentage. The total F and mean F for right leg fat percentage are 3,908.9 and 21.13, 4,610 and 22.27 for the left leg, 5,028.99 and 23.83 for the right arm, 5,031.21 and 23.40 for the left arm, 4,666.94 and 22.88 for trunk, and 4,835.92 and 23.03 for the whole body, respectively.

**Figure 5:**
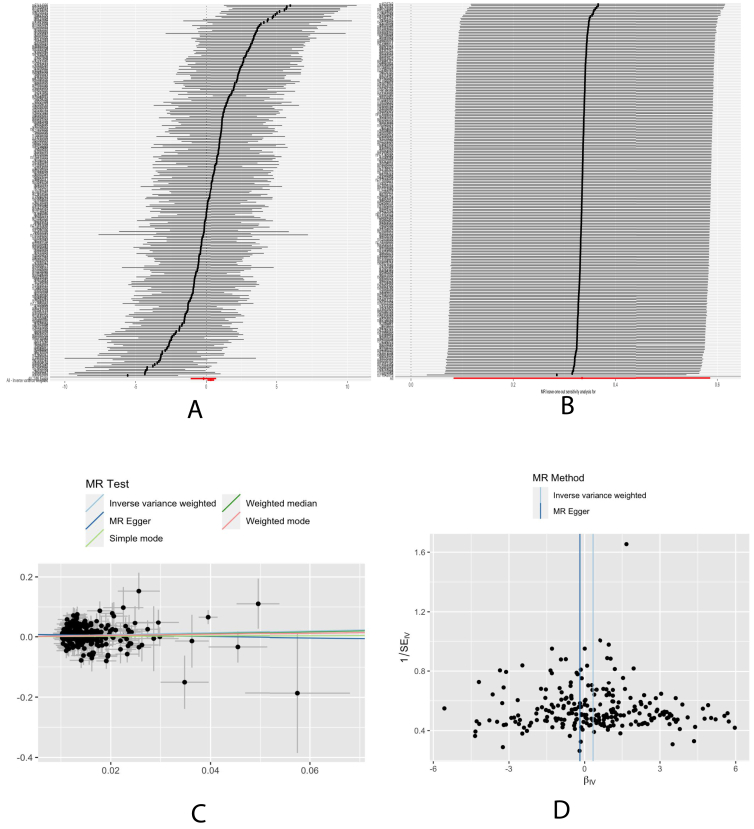
The MR results for the association between appendicular lean mass and CKD risk. Forest plot (A), leave-one-out sensitivity analysis (B), scatter plot (C), and funnel plot (D) of the suggestive causal effect of appendicular lean mass on CKD risk.

According to IVW analysis, genetically predicted right leg fat percentage (odds ratio [OR], 1.91; 95% CI, 1.35–2.70, *P* = 0.0002, FDR=0.0007), left leg fat percentage (OR, 2.02; 95% CI 1.44–2.83; *P* = 0.00004; FDR = 0.0003), and total body fat percentage (OR, 1.59; 95% CI, 1.20–2.12; *P* = 0.001; FDR = 0.003) were causally associated with a higher risk of CKD. However, right arm fat percentage (OR, 1.26; 95% CI, 0.97–1.63; *P* = 0.08; FDR = 0.11), left arm fat percentage (OR, 1.24; 95% CI, 0.96–1.60; *P* = 0.10, HDR = 0.11), and trunk fat percentage (OR, 1.21; 95% CI, 0.95–1.53; *P* = 0.12, HDR = 0.12) were not observed the evidence supporting a causal association with CKD risk. Additionally, genetically predicted appendicular LM was associated with the risk of CKD (Table 3, Fig 5).

**Table 3:** The Suggestive Causal Relationship Between Body Composition and CKD by TSMR

Exposure	Outcome	IVW				MR-Egger			Weighted Median		
		b/OR	95% CI	*P*	FDR	b/OR	95% CI	*P*	b/OR	95% CI	*P*
Right leg fat percentage	CKD	1.91	1.35-2.70	0.0002	0.0007	1.523	0.39-5.99	0.55	1.43	0.89-2.29	0.14
Left leg fat percentage		2.02	1.44-2.83	0.00004	0.0003	1.23	0.33-4.61	0.76	1.55	0.99-2.45	0.06
Right arm percentage		1.26	0.97-1.63	0.08	0.11	1.53	0.63-2.14	0.35	1.46	0.99-2.14	0.06
Left arm percentage		1.24	0.96-1.60	0.10	0.11	1.3	0.57-3.00	0.53	1.24	0.86-1.78	0.25
Trunk percentage		1.21	0.95-1.53	0.12	0.12	0.64	0.26-1.56	0.45	1.15	0.83-1.58	0.40
Body fat percentage		1.59	1.20-2.12	0.001	0.003	1.37	0.47-4.01	0.56	1.74	1.18-2.56	0.005
Appendicular lean mass		0.88	0.79-0.98	0.02	0.03	1.02	0.81-1.31	0.82	0.89	0.75-1.05	0.16

Abbreviations: OR, odds ratio; CI, confidence interval; IVW, inverse-variance weighted; FDR, *P* value corrected for false discovery rate; TSMR, Two-sample Mendelian Randomization.

### Sensitivity Analysis

In the sensitivity analysis, the MR-Egger regression test revealed no horizontal pleiotropy in all primary MR analyses. Nevertheless, the Cochrane Q test detected heterogeneity in right leg fat percentage, left leg fat percentage, right arm fat percentage, left arm fat percentage, trunk fat percentage, and whole-body fat percentage, but no heterogeneity in appendicular muscle mass. Further MR-PRESSO analyses were performed, and the associations of these exposures with CKD became more pronounced after the removal of a few outliers. The leave-one-out analyses demonstrated the results' consistency (Table 4).

**Table 4:** Sensitivity Analysis of the Causal Association Between Body Composition and the Risk of CKD

Exposure	Outcome	MR-IVW			MR-Egger			MR-Egger Intercept			MR-PRESSO
		Q	Q_df	Q_*P* Value	Q	Q_df	Q_*P* Value	Intercept	SE	*P* Value	Global test *P* Value
Right leg fat percentage	Chronic kidney disease	252.37	193	0.003	252.23	192	0.002	0.0028	0.008	0.74	0.01
Left leg fat percentage		254.87	201	0.01	254.13	200	0.006	0.0061	0.008	0.45	0.005
Right arm percentage		219.89	189	0.01	219.65	188	0.06	–0.0031	0.007	0.66	0.04
Left arm percentage		239.92	204	0.04	239.9	203	0.04	–0.0008	0.006	0.91	0.02
Trunk percentage		246.52	184	0.001	243.73	183	0.002	0.0119	0.008	0.15	0.002
Body fat percentage		240.29	183	0.003	240.18	182	0.002	0.0023	0.008	0.78	0.01
Appendicular lean mass		600.41	580	0.27	598.23	579	0.28	–0.004	0.003	0.15	0.31

Abbreviations: CKD, chronic kidney disease; OR, odds ratio; CI, confidence interval; IVW, inverse-variance weighted.

### Reverse MR Analysis

Setting CKD as the exposure, the effect of CKD on the distribution of body fat tissue was evaluated. After removing SNPs with F < 10, we finally filtrated 4 SNPs for CKD, with a total F of 65.89 and a mean F of 13.18. The IVW method revealed no evidence of a causal association between CKD and fat percentages of any body part. However, genetically predicted CKD was negatively associated with appendicular muscle mass (β = –0.02, 95% CI, –0.036 to –0.006, *P* = 0.007) (Table 5).

**Table 5:** The Causal Effect of CKD on Body Composition in Reverse MR

Exposure	Outcome	IVW			MR-Egger			Weighted Median		
		b/OR	95% CI	*P*	b/OR	95% CI	*P*	b/OR	95% CI	*P*
Chronic kidney disease	Right leg fat percentage	0.0015	–0.024 to 0.027	0.91	–0.07	–0.16 to 0.03	0.31	0.01	–0.008 to 0.028	0.27
	Left leg fat percentage	0.0001	–0.025 to 0.252	0.99	–0.07	–0.16 to 0.03	0.29	0.009	–0.009 to 0.026	0.35
	Right arm percentage	–0.003	–0.038 to 0.033	0.89	–0.078	–0.229 to 0.073	0.42	0.003	–0.019 to 0.026	0.79
	Left arm percentage	–0.001	–0.039 to 0.037	0.96	–0.089	–0.246 to 0.068	0.38	0.008	–0.015 to 0.031	0.49
	Trunk percentage	–0.004	–0.049 to 0.041	0.86	–0.106	–0.291 to 0.079	0.38	0.006	–0.021 to 0.033	0.66
	Body fat percentage	–0.002	–0.039 to 0.035	0.92	–0.086	–0.239 to 0.066	0.38	0.007	–0.016 to 0.029	0.57
	Appendicular lean mass	–0.02	–0.036 to –0.006	0.007	0.011	–0.054 to 0.076	0.80	–0.023	–0.041 to –0.006	0.01

### Multivariable MR

In MVMR, we adjusted common risk factors for CKD, including hypertension, type 2 diabetes, and BMI. Genetically predicted fat percentages in both legs continued to exhibit a similar causal association with the risk of CKD as observed in the Two-sample Mendelian Randomization analysis after adjusting for these risk factors. Although the whole-body fat percentage remained positively associated with the risk of CKD after adjusting for BMI and hypertension, genetically predicted body fat percentage was not significantly associated with CKD after adjusting for type 2 diabetes. Moreover, the MVMR did not reveal evidence supporting a causal effect of appendicular muscle mass on the risk of CKD. Similarly, after adjustment for common risk factors for sarcopenia, including malnutrition and physical inactivity, the association between CKD and appendicular muscle mass did not persist (Table 6).

**Table 6:** Multivariable Mendelian Randomization Analysis for CKD and Risk Factors

Exposures	Outcomes	Confounding Factors	IVW			
			OR	95% CI	*P*	FDR
Right leg fat percentage	CKD	T2B	1.59	1.10-2.29	0.01	0.02
		BMI	1.8	1.30-2.48	0.0003	0.001
		Hypertension	1.76	1.18-2.69	0.006	0.02
Left leg fat percentage		T2B	1.66	1.17-2.37	0.005	0.02
		BMI	1.95	1.43-2.65	0.00002	0.0002
		Hypertension	1.82	1.21-2.75	0.004	0.02
Whole-body fat percentage		T2B	1.15	0.86-1.53	0.34	0.34
		BMI	1.35	1.05-1.74	0.02	0.02
		Hypertension	1.46	1.08-1.98	0.01	0.02
Appendicular lean mass		T2B	0.93	0.83-1.05	0.24	0.24
		BMI	0.9	0.80-0.99	0.05	0.15
		Hypertension	0.93	0.92-1.04	0.20	0.24
CKD	Appendicular lean mass	Malnutrition	0.98	0.96-1.00	0.05	0.11
		Physical inactivity	0.98	0.91-1.05	0.51	0.51

Abbreviations: CKD, Chronic kidney disease; T2B, type 2 diabetes; BMI, body mass index.

## Discussion

### Principle Findings

Generally, the major finding of our study is the significant correlation between body composition and kidney outcomes, including RKFD and CKD risk. Both observational and MR analyses provided consistent evidence that appendicular LM and body fat percentage are linked to kidney outcomes, as well as the presence of SO was associated with RKFD risk. Similar findings were also observed in a study suggesting CKD is associated with decreased muscle mass and the coexistence of excess obesity.^34^ However, the epidemiological evidence of the relationship between body composition and the kidney function decline rate of people with normal kidney function has been sparse.

### LM and Kidney Function

Our research uncovered complex relationships between appendicular LM and kidney outcomes. We observed that increased ALMI was associated with a lower risk of RKFD in individuals aged 50-70 years, particularly among overweight participants and those with higher baseline eGFR. Our MR analysis further supported a bidirectional causality between reduced appendicular LM and the risk of CKD. These results align with prior studies showing that sarcopenia-related indicators, such as decreased grip strength and physical inactivity, contribute to CKD development.^35^, ^36^, ^37^ Interestingly, a previous study indicated that lean body mass loss relates to GFR decline in patients with CKD stages 3-5, our results extend this evidence to individuals with normal kidney function.^38^ According to the MVMR results, it is plausible that intermediary risk factors like diabetes and hypertension mediate the effects of ALMI and muscle mass on the decline in kidney function.

### Body Fat Percentage and Its Role

Both our cohort and MR analyses indicated that a higher whole-body fat percentage is associated with kidney function decline, aligning with the understanding that obesity is a common risk factor for CKD.^39^ Although some studies reported an “obesity paradox,” implying that higher BMI is linked to better survival among CKD patients^40^, our findings support the view that fat percentage is a more accurate indicator of risk. This aligns with previous research highlighting the limitations of BMI as a sole measure of obesity.^41^ Our multivariable MR analysis further affirms the relationship between body fat percentage and CKD risk, but this relationship may be partially mediated by type 2 diabetes. In parallel, multivariable Cox regression models in our cohort showed that the direct association between body fat percentage and RKFD was not statistically significant, after adjusting for key confounders such as age, diabetes, and comorbid conditions. This suggests that the relationship may be confounded by some metabolic conditions.

### Body Fat Distribution and SO

We discovered that genetically predicted leg fat percentage was associated with CKD risk, and the RKFD group exhibited higher leg and hip fat percentages. Interestingly, this intriguing observation conflicts with the finding that central obesity was linked to incident CKD^42^ and also with research indicating that leg FM and hip circumference may positively contribute to cardiovascular risk factors.^43^ This may reflect changes in fat-to-muscle ratios were the key factors contributing to metabolic risk because there is no discernible difference in FM between the 2 groups in our cohort study. As a result, we further examined SO, which represents the coexistence of low muscle mass and high fat percentage. We observed a higher incidence of SO in the RKFD group, and this association remained significant after adjusting for confounders in the Cox regression analysis, suggesting that SO may serve as a risk factor for a rapid kidney function decline and incident CKD.

### Potential Mechanisms

First, skeletal muscle insulin resistance is a primary defect in type 2 diabetes as the skeleton muscle is the predominant site of insulin-mediated glucose uptake^44^, highlighting the protective metabolic role of maintaining LM. In contrast, increased body fat is associated with elevated inflammatory biomarkers.^45^ Chronic, low-grade inflammation has been recognized as a major pathogenic mechanism connecting CKD and obesity.^46^ Additionally, ectopic lipid accumulation in the kidneys may contribute to structural and functional damage in key kidney cells, further impairing kidney function.^47^

### Clinical Implications

Our findings suggest that routine assessment of body composition could aid in the early identification of individuals with normal baseline kidney function at risk for RKFD, and further risk of developing CKD. Tools such as bioelectrical impedance analysis, DEXA, or CT now have been used in many clinical settings^48^ and can be further used for risk stratification methods for kidney outcomes. Furthermore, our results support the potential benefit of interventions aimed at increasing muscle mass and reducing fat percentage, such as resistance training, nutritional guidance, and physical activity. These interventions could possibly mitigate the kidney function decline rate and further delay CKD progression.

### Strengths and Limitations

Our study has several strengths, the primary one being the integration of MR analysis and cohort study. The MR design minimizes residual confounding and reverses causality, bolstering the causal inferences made regarding the associations between exposure and CKD. To improve the generalizability of our findings, we conducted a observational cohort study in a Chinese population and included European ancestry data in the MR analysis. The consistent direction of associations across both populations provides supportive evidence for a potential link between body composition and risk of kidney outcomes. However, given the differences across populations, further research in more diverse and multiethnic cohorts is warranted to confirm the broader applicability of these findings.

However, there are still some limitations in the current study. First, further investigation to confirm the association between body composition and incident CKD in observational study may needed. Second, the presence of sample overlap for certain exposures in our analysis may introduce some bias toward observational estimates. Despite this, the robustness of our findings is supported by high *F* statistics in our MR analysis, indicating a minimal bias resulting from sample overlap.

### Conclusion

In conclusion, in the cohort study, we discovered that SO was associated with higher RKFD risk and an increase in ALMI was related to lower risk of RKFD. The subgroup analysis suggested that among participants who were overweight (BMI ≥ 24 kg/m^2^) and had baseline eGFRs > 90 mL/min/1.73 m^2^, there was a significant correlation between ALMI and RKFD. Our MR study provides strong proof of causal associations between body composition and CKD risk. Notably, higher right/left leg fat percentages, increased whole-body fat percentages, and lower appendicular muscle mass are significant contributors to CKD risk. MVMR also highlight the potential influence of mediating factors such as type 2 diabetes, physical activity, and nutrition.
