# Supplementary material for: Body Composition and Kidney Outcomes: A Cohort Study of Rapid Kidney Function Decline and a Mendelian Randomization Analysis of CKD Incidence
Source: Kidney Med. 2025 Aug 14;7(10):101087. doi: 10.1016/j.xkme.2025.101087 (PMC12495164; doi:10.1016/j.xkme.2025.101087)
Supplement: Supplementary File (PDF) — Table S1. [file mmc1.pdf]

Table S1. Association between body composition and RKFD in univariate Cox regression

Variables	HR	CI	*p*-value
whole body fat percentage	1.088	1.002-1.181	0.045*
FMI	1.272	0.974-1.662	0.077
ALMI	0.493	0.323-0.753	0.001*
abdominal fat percentage	1.008	0.918-1.106	0.874
hip fat percentage	1.081	1.009-1.158	0.028*
left arm fat percentage	1.049	0.995-1.105	0.075
right arm fat percentage	1.052	0.999-1.108	0.054
trunk fat percentage	1.057	0.967-1.155	0.225
left leg fat percentage	1.075	1.012-1.142	0.019*
right leg fat percentage	1.073	1.012-1.139	0.019*
High fat percentage	1.993	0.780-5.091	0.15
Sarcopenic obesity	3.618	1.550-8.441	0.003*

Abbreviation: FMI: Fat Mass Index; ALMI: Appendicular Lean Mass Index
